# Changes in trends and outcomes of eclampsia: a success story from Qatar

**DOI:** 10.5339/qmj.2019.10

**Published:** 2019-09-20

**Authors:** Hussain A Sharara, Nissar Shaikh, Firdous Ummunnisa, Naseera Aboobacker, Halima Al Tamimi

**Affiliations:** ^1^Department of Obstetrics & Gynecology, Al-Khor Hospital, Hamad Medical Corporation, Qatar; ^2^Surgical Intensive Care Unit, Hamad General Hospital, Hamad Medical Corporation, Doha, Qatar; ^3^Women's Wellness and Research Center, Hamad Medical Corporation, Doha, Qatar

**Keywords:** antenatal follow-up, seizures, eclampsia, hypertension, proteinuria, magnesium sulfate, posterior reversible encephalopathy syndrome

## Abstract

**Background:** Preeclampsia and eclampsia are two hypertensive disorders of pregnancy that significantly contribute to higher morbidity and mortality rates. Eclampsia is the occurrence of seizures in patients with preeclampsia without any previous history of seizure disorders. The incidence and prevalence of eclampsia varies, and there is scarce literature about the prevalence and trends of eclampsia in the Arabian Gulf countries. This study aimed to identify the incidence and changing trends of eclampsia in Qatar. **Patients and Methods:** This retrospective study was conducted at Hamad Medical Corporation, the only tertiary heathcare center in Qatar, and included all patients admitted with eclampsia from 1979 to 2017. The demographic data, maternal age, parity, gestational age, antenatal visits, timing of seizures, mode of delivery, treatment, maternal morbidity, and mortality were recorded. The study period was divided into the initial period of 1979–1988, 1991–2009, and the recent period of 2010–2017. Data analysis was performed using chi-square tests to identify trends among the three different periods. A *p* value of ≤ 0.05 (two-tailed) was considered statistically significant. **Results:** A total of 151 patients with eclampsia were admitted with an increasing incidence of eclampsia over time. There was a statistically significant improvement in antenatal follow-up and an increase in the maternal age of patients with eclampsia (*p* = 0.001). The incidence of eclampsia without proteinuria increased significantly over time (*p* = 0.03). Postpartum eclampsia was more common (*p* = 0.002). Labetalol was the most frequently used antihypertensive agent (*p* = 0.001), and magnesium sulfate has been increasingly used as an anticonvulsant agent (*p* = 0.001). The rate of maternal morbidity was decreasing, and in the recent period, posterior reversible encephalopathy syndrome was becoming a common comorbidity in patients with eclampsia. Maternal mortality displayed significant improvement, reaching 0% in the recent study period (*p* = 0.02). Perinatal mortality likewise displayed a decreasing trend and reached 3.17% in the recent period. **Conclusion:** The incidence of eclampsia is increasing in Qatar. The antenatal care of patients with eclampsia has improved significantly. The medical management of patients with eclampsia has also drastically improved, leading to a significant decrease in maternal mortality and improvement in perinatal outcomes.

## Background

Eclampsia is not a new disease; it has been recognized since the days of Hippocrates and still exists. In combination with preeclampsia, eclampsia contributes to half of the mortalities from hypertensive disorders of pregnancy. Preeclampsia is the occurrence of hypertension with a blood pressure of >140/90 mm Hg on two separate occasions 4 hours apart or of 160/110 mm Hg on one occasion in combination with the presence or even absence of proteinuria and the exclusion of molar pregnancy.^1^ Most patients with preeclampsia progress into eclampsia. Eclampsia is defined as the onset of new seizures in patients with preeclampsia who are not known to have any convulsive disorders.^1,2^ The incidence of eclampsia varies significantly in developed and developing countries from 1.6–10 to 6–157 per 10,000 deliveries, respectively.^3,4^ Preeclampsia and eclampsia can lead to vital end-organ damage, primarily the kidneys, liver, heart, and brain, which can be potentially life threatening.^5^ These hypertensive disorders of pregnancy are also a constant threat to the fetus and cause fetal growth retardation, congenital heart disease, stillbirth, and preterm delivery.^5^ There is a lack of information and scarce literature on the trends of eclampsia in the Arabian Gulf countries.

The aim of this study was to identify the incidence, trends, and outcomes of eclampsia in Qatar.

## Patients and methods

This retrospective study was conducted at Hamad Medical Corporation, the only tertiary healthcare facility in Qatar. After receiving permission from the medical research department (Research Proposal Number: 10044/10) of the institution, all patients admitted with eclampsia from 1979 to 2017 (data for 1989 and 1990 could not be collected because of data unavailability) were retrospectively reviewed, and information on demographic characteristics, maternal age, parity, gestational age, antenatal visits, presence of hypertension, proteinuria, timing of seizures, mode of delivery, anticonvulsant and antihypertensive treatment, and maternal morbidity and mortality was recorded.

Eclampsia was diagnosed in all these patients by the occurrence of seizures during pregnancy, intrapartum, and up to 10 days postpartum. Patients with other etiologies for seizures such as brain lesions and those who were known to have convulsive disorders were excluded from the study. The study period was divided into three periods to determine eclampsia trends: the initial period of 1979–1988, 1991–2009 and the recent period of 2010–2017.

All patients were treated with standard care that aimed to control the seizures, provide safe delivery of the fetus, and treat hypertension and other maternal complications. All patients were treated in the intensive care setup at Hamad Medical Corporation. Data were entered in the SPSS program (version 19; IBM Corp., Armonk, NY, USA), and chi-square tests were performed to identify trends among the three different periods. A *p* value of ≤ 0.05 (two-tailed) was considered statistically significant.

## Results

There were a total of 463,016 deliveries during the study period, with a range of 9452–24,322 deliveries per year. The number of deliveries increased each year. There was a total of 151 patients admitted with eclampsia (Table 1). The incidence of eclampsia ranged from 0 to 12 cases per year, and an overall prevalence of 3.26 cases of eclampsia per 10,000 deliveries was observed, with an increasing trend (Table 1). Although the incidence of eclampsia was increasing, this increase was not statistically significant (*p* = 0.13).

Table 2 shows the descriptive data. Most of the patients with eclampsia were expatriate females (n = 14, 77.78%; n = 42, 60%; and n = 50, 79.37% in 1979–1988, 1991–2009, and 2010–2017, respectively); there was no significant difference in the proportion of expatriates among the periods (*p* = 0.24). Age differed significantly over time (*p* = 0.001). In the initial period of the study, 15 patients 83% of the patients with eclampsia were aged < 25 years, but by the recent period, 47 patients 74% of the patients with eclampsia were aged >25 years. There was also a significant difference in the trend of patients with eclampsia receiving antenatal follow-up (*p* = 0.001); the antenatal visit pattern improved in the recent period compared with the initial period, with n = 13, 72.23%; n = 21, 30.02%; and n = 6, 11.5% of patients not followed up in the respective periods. There were no significant changes in the occurrence of eclampsia and gestational age over time (*p* = 0.64); most of the patients developed eclampsia after 37 weeks of gestation. Primiparous women were more commonly affected by eclampsia (n = 13, 72.2%; n = 46, 65.7%; and n = 34, 53.96% of patients in the respective periods), and the difference in trends was insignificant (*p* = 0.64). Eclampsia occurred in the antepartum period in 15 (83.33%) and 31 (44.3%) patients in the 1979–1988 and 1991–2009 periods, respectively, whereas in the recent period (2010–2017), postpartum eclampsia was more frequent (n = 29, 46.04%). This difference was statistically significant (*p* = 0.002).

The most common mode of delivery of the fetus remained as the lower segment cesarean section (LSCS) (n = 15, 83%; n = 45, 64%; and n = 46, 73% in the respective periods) (Table 2), and this trend remained statistically insignificant (*p* = 0.75). Most of our patients had proteinuria (100%, 74%, and 69% in the respective periods) (Table 2), but the occurrence of eclampsia without proteinuria is increasing, and this trend was statistically significant (*p* = 0.03). There were more patients with eclampsia with mild hypertension in the 1991–2009 period, but this proportion decreased in the recent period 24 (30%) and 4 (7.7%) patients in 1991–2009 and 2010–2015 periods, respectively]. In the initial period of the study, hydralazine was the only antihypertensive medication used, but more recently an increasing number of patients are treated with labetalol (n = 53, 84.13%); this trend was statistically significant (*p* = 0.01) (Table 2).

During the initial period of the study, all patients were treated with benzodiazepines for seizures; however, the use of benzodiazepines has since decreased, whereas magnesium sulfate was used more frequently in the 1991–2009 period (n = 39, 55.71% vs. n = 31, 44.28% in 1991–2009 vs. 2010–2017). In the recent period, all patients with eclampsia received magnesium sulfate with or without benzodiazepines and anticonvulsants to control the convulsions; these changes in treatment trends were statistically significant (*p* = 0.01) (Table 2).

Table 3 shows the morbidities of the patients with eclampsia. During the initial period, the most common complications were cerebrovascular accidents (n = 2, 11.12%) and peripartum hemorrhage (n = 2, 11.2%); additionally, two (11.12%) patients developed acute kidney injury, and one (5.6%) patient had pneumonia. HELLP (hemolysis, elevated liver enzymes, and low platelet count) syndrome was not defined during this period, but none of these patients had thrombocytopenia and/or elevated liver enzymes. Two patients had cerebrovascular accidents. Seven (38.8%) patients did not develop any complications. From 1991 to 2009, 46 (67%) patients with eclampsia did not have any complications. The most common complication was HELLP syndrome (10 patients, 14.3%). Peripartum hemorrhage occurred in eight (11.4%) patients. Two (2.84%) patients had cerebrovascular accidents/posterior reversible encephalopathy, and one (1.42%) patient had acute kidney injury. In the recent period, 39 (61.91%) patients with eclampsia did not have any complications. The most common complications were posterior reversible encephalopathy syndrome (PRES) (n = 17, 26.99%) and peripartum hemorrhage (5 patients, 7.94%). HELLP syndrome occurred in only four (6.34%) patients; thus, there has been a decrease in the occurrence of HELLP syndrome in patients with eclampsia. Two (3.18%) patients with eclampsia developed pneumonia, and two (3.18%) patients with PRES had transient blindness. One (1.58%) patient with PRES had pulmonary edema. The occurrence of acute kidney injury in patients with eclampsia was significantly reduced over time (*p* = 0.01), and there has been a significantly increasing trend of PRES in patients with eclampsia (*p* = 0.002) (Table 3).

**Figure 1.**
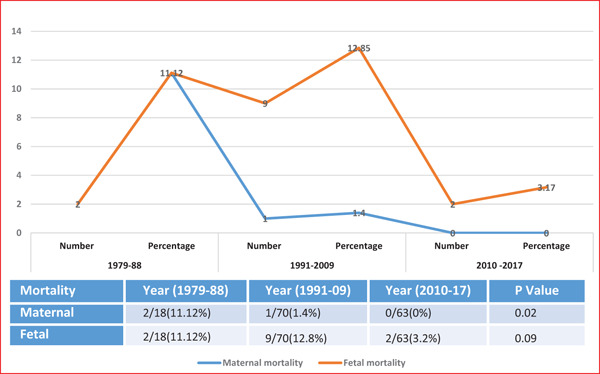
Maternal and fetal mortality.

Maternal mortality significantly decreased during the study period (*p* = 0.02); although perinatal mortality also showed a decreasing trend, this change was not statistically significant (Figure 1). During the initial study period, both patients with cerebrovascular accidents had died, giving a mortality rate of 11.2%. From 1991 to 2009, one patient died who had a combination of severe thrombocytopenia, HELLP syndrome, and PRES, giving a mortality rate of 1.4%. Although there was an increased incidence of cerebral complications (PRES) in the recent period, none of the patients had a fatal outcome, bringing the mortality rate down to 0% (Figure 1). Likewise, perinatal mortality decreased in the recent period to 3.17% (Figure 1).

## Discussion

Eclampsia is a life-threatening seizure disorder that occurs during pregnancy and the postpartum period; for differential diagnosis, other causes of convulsions such as known epilepsy, cerebral infections, and brain tumors should be excluded.^1–3^ Eclampsia is therefore considered an obstetrical emergency.

The incidence of eclampsia has remained fairly stable in Qatar at approximately 3/10,000 deliveries with a slightly increasing trend. There are no previous studies about the trends of eclampsia in this region. The reported eclampsia incidences are higher in Saudi Arabia and Egypt (5.6/1000 and 115.2/1000, respectively).^6,7^ Recent statistics from the United States of America (USA) also show an increasing trend of eclampsia.^8^ Expatriate patients seem to have a higher occurrence of eclampsia than the local patients. This may be due to the large number of expatriate population in Qatar.

In our study, eclampsia was more common among primiparous women. The regional and international literatures have also reported similar findings.^6,7,9^ Eclampsia was more common during the postpartum period in our patients and may be related to increased maternal age. This is in contrast to the more frequent occurrence of eclampsia in the antepartum period in other regional countries.^6,9,10^ In one study, eclampsia most frequently occurred during the antepartum period (59%), followed by the postpartum period (21%) and the intrapartum period (20%).^11^

The number of patients with eclampsia with mild hypertension and without proteinuria was noted to significantly increase. This has also been reported in the literature, although there is no literature from this specific region about eclampsia without proteinuria. Furthermore, 38% of patients in the United Kingdom (UK) and 16% in the USA had eclampsia without hypertension and proteinuria.^12,13^

Our study showed an increasing trend in maternal age in patients with eclampsia. A tertiary care hospital from this region also found increased maternal age in patients with eclampsia.^7^ Lamminpaa et al.,^14^ from Finland published a similar observation of advanced maternal age exhibiting more preeclampsia and eclampsia compared with younger women.

Our trend of constant improvement in the antenatal follow-up of patients with eclampsia may have had an impact on the improved outcome of these patients. The literature from regional countries and the Indian subcontinent still shows poor and much lower antenatal follow-up rates.^6,7,9,10^

Recently, eclampsia has been reported more commonly after 37 weeks of gestation, whereas during the 1991–2009 period, there was a higher incidence of eclampsia before 37 weeks of gestation. The regional literature reports eclampsia to be common after 34 weeks of gestation.^7^ LSCS was the most frequent mode of fetus delivery in all phases of our study; similar findings have been reported from literature of the region, with an Indian study showing an increase in the rate of LSCS in these patients.^15^ In a randomized controlled trial, Seal et al.,^16^ concluded that after 32 weeks of gestation, with a favorable Bishops Score, the induction of labor should be encouraged. Their randomized controlled trial succeeded in having a vaginal delivery rate of 70% in patients with eclampsia.^16^

The present study shows a significant change in the use of anticonvulsant therapy over time. Initially, only benzodiazepines were used for seizure control, and patients undergoing this treatment had a higher incidence of recurrent fits. However, beginning in the mid-90s, magnesium sulfate was used, and the occurrence of recurrent seizures decreased. Duley et al.,^17^ concluded in their review that magnesium sulfate is safer, does not require cardiovascular monitoring, and is more effective than benzodiazepines and lytic cocktails for the treatment and prevention of recurrent seizures in patients with eclampsia.^17,18^ In the recent period of this study, all our patients received magnesium sulfate. Thus, the use of magnesium sulfate is increasing in the region, with one regional country reporting that all patients with eclampsia were treated with magnesium sulfate.^7^

Persistent hypertension is correlated with increased morbidity and mortality in patients with eclampsia.^2,3^ There was a significant change in the use of antihypertensive medication in our study. In the initial period, all patients with eclampsia received hydralazine infusion to control hypertension; however, the use of labetalol infusion has increased recently, and a few patients have received a combination of these medications. In a meta-analysis comparing hydralazine and labetalol, Magee et al.,^19^ showed that labetalol was a better choice because it had fewer adverse effects.

The maternal complications in our patients with eclampsia have changed in an interesting manner over time. During the initial decade, the most common maternal morbidity was cerebrovascular accidents, followed by peripartum hemorrhage. From 1991 to 2009, HELLP syndrome was the major complication in patients with eclampsia, followed by peripartum hemorrhage. One patient with eclampsia died during this period due to the combined complications of HELLP syndrome and PRES. In the recent period of our study, the most common morbidity was cerebral complications, namely PRES (26.99% of patients), followed by peripartum hemorrhage (7.9%). In a Saudi eclampsia study, the main maternal morbidity was HELLP syndrome,^6^ whereas an eclampsia study from Kuwait reported that abruptio placentae was the most frequent maternal morbidity,^10^ and an Egyptian study found the HELLP syndrome to be the most common maternal morbidity.^7^ In an African study, the most common complication was cerebrovascular accident (15%), followed by HELLP syndrome (4.2%).^20^ In an Indian study by Pannu et al.,^21^ the most common maternal complication was PRES (16.8%), followed by HELLP syndrome (13.2%) and acute renal failure (12%). In our study, there was a decrease in the occurrence of HELLP syndrome, and no patients with eclampsia had acute kidney injury or pneumonia in the recent period (Table 3).

There has been a significant decreasing trend in maternal mortality that reached 0% in the recent period. This reduction was achieved mainly due to increased awareness, increased antenatal follow-up, and supportive care by multidisciplinary teams. The maternal mortality rate in developed countries ranges from 0% to 1.8%, whereas it can reach up to 14% in developing countries.^22^ The eclampsia maternal mortality rate in studies from Saudi Arabia and Kuwait was reported to be 0%, whereas from an Egyptian study, it was 1.6%. The main reason for the higher mortality in Egypt was the lack of antenatal follow-up and acute care settings.^6,7,10^

Perinatal mortality has also displayed a decreasing trend, reaching 3.17% in recent years. The higher perinatal mortality rate during the 1991–2009 period may be related to higher maternal morbidity from HELLP syndrome. Regional countries have reported significantly higher perinatal mortality rates ranging from 3.3% to 16%.^6,7,10^ The perinatal mortality rates from eclampsia in the USA and UK range from 5.6% to 11.8%.^13,23^

There are some limitations to our study. This was a retrospective single-center study. Also, data for two years is missing, and the changes in demographics and a larger expatriate population make comparisons difficult.

## Conclusion

There is an increasing trend of eclampsia in Qatar, but there has been significant improvement in the antenatal follow-up of patients with eclampsia. The age of patients with eclampsia is also increasing. Moreover, postpartum eclampsia has been significantly increasing in our population. Eclampsia without proteinuria is also increasing in our patient population. LSCS remains the most common mode of fetus delivery. There has been a significant change in the use of anticonvulsants, with magnesium sulfate being more frequently used to control seizures in these patients. Labetalol infusion is increasingly used as an antihypertensive agent in our eclampsia population. There have been changes in maternal morbidity patterns as well: in the initial period, cerebrovascular accidents and peripartum hemorrhage were more frequent, whereas HELLP syndrome was more common in the 1991–2009 period; in the recent study period, posterior reversible encephalopathy was the most common maternal morbidity reported.

Despite the increase in eclampsia, our maternal morbidity and mortality rates are displaying decreasing trends. Maternal mortality has reached 0% and has remained at this level for 7 years, and the perinatal mortality rate is on the decline as well.

## Figures and Tables

**Table 1:** Incidence of eclampsia over the study period

Year	Number of deliveries	Eclampsia	Incidence	*P*-value

1979–88	76,000	18	2.3/10,000	0.13

1991–2009	224,809	70	3/10,000	

2010–2017	162,207	63	3.88/10,000	

Total	463,016	151	3.26/10,000	


**Table 2:** Descriptive data showing relation of eclampsia with demographic, gestation and treatment parameters.

Year	1979–88		1991–2009		2010–2017		

**Eclampsia and nationality**	Number (n)	Percentage (%)	Number (n)	Percentage (%)	Number (n)	Percentage (%)	*P*-value

Qatari	4	22.23	28	40	13	20.63	0.24

Non Qatari	14	77.78	42	60	50	79.37	

**Eclampsia and age**							

< 25 years	15	83.33	47	66.7	16	25.38	0.001

>25years	3	16.66	23	33.3	47	74.61	

**Eclampsia and antenatal visits**							

None	13	72.23	21	30.02	6	11.5	0.001

< 10	3	16.67	16	22.84	13	20.64	

>10 Regular	2	11.12	33	47.14	44	69.85	

**Eclampsia and gestational age**							

< 37 weeks	6	33.33	38	54.2	30	47.62	0.64

>37 weeks	12	66.66	32	45.7	33	52.38	

**Eclampsia and gravida**							

Primiparous	13	72.22	46	65.7	34	53.96	0.21

Multiparous	7	38.88	24	34.3	29	46.04	

**Eclampsia and timing of fit**							

Antepartum	15	83.32	31	44.3	24	38.09	0.002

Intrapartum	1	5.5	17	24.3	10	15.87	

Postpartum	2	11.12	22	31.4	29	46.04	

**Eclampsia and mode of delivery**							

Lower section caesarean section	15	83.33	45	64.3	46	73.02	0.75

Normal delivery	3	16.66	17	24.2	12	19.05	

Vaginal assisted delivery	0	0	8	11.5	5	7.93	

**Eclampsia and proteinuria**							

Present	18	100	52	74	44	69.84	0.03

Absent	0	0	18	26	19	30.16	

**Eclampsia and antihypertensive**							

None	0	0	24	30	4	6.34	0.001

Labetalol	0	0	15	23	53	84.13	

Hydralazine	18	100	34	48	2	3.17	

Labetalol + hydralazine	0	0	0	0	4	6.34	N/A*

**Eclampsia and anticonvulsant**							

Magnesium sulphate	0	0	39	55.71	46	73.02	0.001

Benzodiazepine	18	100	31	44.28	0	0	

Magnesium sulphate + benzodiazepine	0	0	0	0	19	30.16	N/A*

Magnesium sulphate + anticonvulsant	0	0	0	0	3	4.77	N/A*


*Not applicable.

**Table 3:** Maternal morbidities.

Year	1979–88		1991–2009		2010–2017		

**Morbidity**	Number (n)	Percentage (%)	Number (n)	Percentage (%)	Number (n)	Percentage (%)	P-value

None	14	79.78	46	67	39	61.91	

HELLP syndrome*	0	0	10	14.3	4	6.34	0.91

Peripartum hemmorrhage	2	11.2	8	11.44	5	7.94	0.55

Acute kidney injury	2	11.12	1	1.42	0	0	0.01

Pneumonia	1	5.6	2	2.84	0	0	0.10

Pulmonary edema	0	0	0	0	2	3.18	0.13

Cerebrovascular accident (**CVA**)/Posterior Reversible Encephalopathy Syndrome (**PRES**)	2	11.12	2	2.84	17	26.99	0.002


*HELLP (Haemolysis, elevated liver enzymes low platelet) syndrome*
